# Resuscitative endovascular balloon occlusion of the aorta (REBOA) in patients with major trauma and uncontrolled haemorrhagic shock: a systematic review with meta-analysis

**DOI:** 10.1186/s13017-021-00386-9

**Published:** 2021-08-12

**Authors:** Greta Castellini, Silvia Gianola, Annalisa Biffi, Gloria Porcu, Andrea Fabbri, Maria Pia Ruggieri, Carlo Coniglio, Antonello Napoletano, Daniela Coclite, Daniela D’Angelo, Alice Josephine Fauci, Laura Iacorossi, Roberto Latina, Katia Salomone, Shailvi Gupta, Primiano Iannone, Osvaldo Chiara, the Italian National Institute of Health guideline working group on Major Trauma, Nino Stocchetti, Elvio De Blasio, Gaddo Flego, Massimo Geraci, Giulio Maccauro, Antonio Rampoldi, Federico Santolini, Claudio Tacconi, Gregorio Tugnoli

**Affiliations:** 1 IRCCS Istituto Ortopedico Galeazzi, Unit of Clinical Epidemiology, Milan, Italy; 2 National Centre for Healthcare Research and Pharmacoepidemiology, Department of Statistics and Quantitative Methods, University of Milano-Bicocca, Milan, Italy; 3 Unit of Biostatistics, Epidemiology and Public Health, Department of Statistics and Quantitative Methods, University of Milano-Bicocca, Milan, Italy; 4 Emergency Department, AUSL della Romagna, Forlì, Italy; 5 Emergency Department, AO San Giovanni Addolorata, Rome, Italy; 6 Department of Anesthesiology, Intensive Care and Pre-Hospital Emergency Services, Maggiore Hospital Carlo Alberto Pizzardi, Bologna, Italy; 7 Istituto Superiore di Sanità, Centro Eccellenza Clinica, Qualità e Sicurezza delle Cure, Rome, Italy; 8 University of Maryland, Adams Cowley Shock Trauma Center, Baltimore, MD USA; 9 Department of Pathophysiology and Transplantation, University of Milan, Milan, Italy; 10 General Surgery and Trauma Team, ASST Grande Ospedale Metropolitano Niguarda, University of Milan, Milano, Piazza Ospedale Maggiore, Milan, Italy

**Keywords:** Systematic review, Resuscitative Endovascular Balloon Occlusion of the Aorta, Major trauma haemorrhage, Resuscitative thoracotomy

## Abstract

**Background:**

Multiple studies regarding the use of Resuscitative Endovascular Balloon Occlusion of the Aorta (REBOA) in patients with non-compressible torso injuries and uncontrolled haemorrhagic shock were recently published. To date, the clinical evidence of the efficacy of REBOA is still debated. We aimed to conduct a systematic review assessing the clinical efficacy and safety of REBOA in patients with major trauma and uncontrolled haemorrhagic shock.

**Methods:**

We systematically searched MEDLINE (PubMed), EMBASE and CENTRAL up to June 2020. All randomized controlled trials and observational studies that investigated the use of REBOA compared to resuscitative thoracotomy (RT) with/without REBOA or no-REBOA were eligible.

We followed the PRISMA and MOOSE guidelines. Two authors independently extracted data and appraised the risk of bias of included studies. Effect sizes were pooled in a meta-analysis using random-effects models. The quality of evidence was assessed using the Grading of Recommendations Assessment, Development and Evaluation methodology. Primary outcomes were mortality, volume of infused blood components, health-related quality of life, time to haemorrhage control and any adverse effects. Secondary outcomes were improvement in haemodynamic status and failure/success of REBOA technique.

**Results:**

We included 11 studies (5866 participants) ranging from fair to good quality. REBOA was associated with lower mortality when compared to RT (aOR 0.38; 95% CI 0.20–0.74), whereas no difference was observed when REBOA was compared to no-REBOA (aOR 1.40; 95% CI 0.79–2.46). No significant difference in health-related quality of life between REBOA and RT (*p* = 0.766). The most commonly reported complications were amputation, haematoma and pseudoaneurysm. Sparse data and heterogeneity of reporting for all other outcomes prevented any estimate.

**Conclusions:**

Our findings on overall mortality suggest a positive effect of REBOA among non-compressible torso injuries when compared to RT but no differences compared to no-REBOA. Variability in indications and patient characteristics prevents any conclusion deserving further investigation. REBOA should be promoted in specific training programs in an experimental setting in order to test its effectiveness and a randomized trial should be planned.

**Supplementary Information:**

The online version contains supplementary material available at 10.1186/s13017-021-00386-9.

## Background

Haemorrhage from non-compressible torso injuries is a leading cause of death in military and civilian trauma [1]. To control exsanguinating bleeding from non-compressible torso injuries, a damage control approach should be used. A variety of damage control surgery techniques have been developed to limit blood loss, control contamination and preserve one’s physiology such as abdominal packing, non-essential organ removal, extra-peritoneal packing, stapler resection of the bowel, vascular shunts and interventional radiology with embolization procedures.

Resuscitative thoracotomy is commonly used in patients in extremis or cardiac arrest for open cardiac massage and aortic cross-clamping [2, 3]. Resuscitative Endovascular Balloon Occlusion of the Aorta (REBOA) is a technique for temporary cessation or limitation of blood flow through the aorta, which may be used as a bridge until definitive control of the bleeding by endovascular procedures or surgery is performed [4]. After emergency room extended focused assessment sonography for trauma (E-FAST) and chest and pelvis x-ray, the balloon can be inflated in the descending thoracic aorta (zone 1) to reduce blood flow below the diaphragm or in the abdominal aorta below the renal arteries (zone 3) to stop bleeding from the pelvis and lower extremities.

The application of a REBOA has been suggested in the following cases: (i) in zone 1 for imminent traumatic cardiac arrest for probable haemorrhagic cause [5]; (ii) in zone 1 for severe haemorrhagic shock due to abdominal and/or pelvic injuries [6, 7]; (iii) in zone 3 for severe pelvic fracture [7, 8] or to control junctional bleeding from the groin end lower extremities; (iv) in zone 1 for penetrating thoracic trauma, according to an algorithm proposed in 2020 [9].

In recent years, REBOA has received a lot of attention for its applicability and promise in adult major trauma settings. It is a less invasive method of haemodynamic control in severe haemorrhagic settings relative to other damage control techniques. Survival benefits between REBOA and non-REBOA in severe abdominal-pelvic haemorrhage and between REBOA and resuscitative thoracotomy (RT) in imminent cardiac arrest for haemorrhage are controversial. All procedures may lead to unintended adverse effects [10] and this underlines the need for shared indications for the use.

The aim of this systematic review was to explore to the best of current knowledge if REBOA is clinically safe and effective in the management of major exsanguination from torso injuries due to trauma.

## Methods

We conducted a systematic review to support the major trauma integrated management guideline panel of the Italian National Institute of Health (Istituto Superiore di Sanità) in formulating recommendations [11]. Following the GRADE-ADOPOLMENT methodology [12] and in accordance with the standards defined by the Sistema Nazionale Linee Guida (SNLG) [13], the multidisciplinary panel decided to develop a “de novo” question addressing the efficacy of Resuscitative Endovascular Balloon Occlusion of the Aorta (REBOA) on patients with major trauma. The clinical question addressed in this systematic review was: Is Resuscitative Endovascular Balloon Occlusion of the Aorta (REBOA) clinically effective in the Management of major exsanguination in trauma?

### Registered protocol

The protocol of the present systematic review is stored at the following link: https://osf.io/ntxvj/. We conducted the systematic review following the Preferred Reporting Items for Systematic Reviews and Meta-Analyses (PRISMA) statement and the Meta-Analysis of Observational Studies in Epidemiology (MOOSE) guideline [14, 15].

### Inclusion criteria

Randomized controlled trials (RCT) and/or observational studies were included if they met the following criteria: (1) *population*: children, young people and adults experiencing major trauma, blunt or penetrating; (2) *intervention*: REBOA; (3) *comparison*: RT (with/without REBOA) or no REBOA intervention; (4) *setting*: pre-hospital, emergency department and operating room resuscitation phase. Studies including patients with trauma resulting from burns were excluded.

### Outcome measures and follow-up assessment

Primary outcome measures selected for the analyses were as follows: (i) 24-h mortality, 30 days to 12 months mortality; (ii) volume of infused blood components; (iii) health-related quality of life (e.g. Discharge Glasgow Coma Scale); (iv) adverse effects (e.g. amputation); (v) time to haemorrhage control. Secondary outcomes were as follows: (vi) improvement in haemodynamic status and (vii) failure/Success of REBOA technique.

### Search strategy

Two professional librarians interviewed the following electronic databases: MEDLINE (PubMed), EMBASE (Elsevier, EMBASE.com) and CENTRAL up to June 9, 2020, with language restricted to English, Italian, Spanish, French, German using the search strategy outlined in Supplement A. We checked the reference lists of all studies included and of any systematic reviews we identified during the search process (including grey literature and conference proceedings). We also searched for ongoing trials (i.e. clinical trials.gov).

### Study selection and data extraction

Two independent authors (SG, GC) screened titles and abstracts by the search strategy. Following the first phase, they independently assessed the full text of potentially relevant studies for inclusion. Any disagreement was solved by a discussion with one of the authors (OC). A standardized data collection form was used to extract the following information: (i) study characteristics: study design, setting, countries and settings, funding; (ii) participant’s characteristics, sample size and type of trauma; (iii) intervention type and outcomes. The authors of the selected studies were contacted if the reported data were not reported in detail or were incomplete. We hand searched potential references from lists of included studies.

### Internal validity

The internal validity of the included studies was assessed using the Cochrane Risk of Bias (RoB) tool for RCTs [16] and the Newcastle-Ottawa scales [17] for observational studies. The following domains of the Cochrane RoB tool were appraised: selection bias (random sequence generation and allocation concealment), performance bias (blinding of participants and personnel), detection bias (blinding of outcome assessment), attrition bias (incomplete outcome data) and reporting bias (selective reporting) (58). Each domain was classified as “high”, “low” or “unclear” RoB if the study did not provide sufficient information to be classified.

In the Newcastle-Ottawa scales, the following domains were appraised: selection, comparability, outcome. Thresholds for converting the Newcastle-Ottawa scales to Agency for Healthcare Research and Quality standards (good, fair and poor) were adapted. Two reviewers (SG, GC) independently evaluated the methodological quality of the included studies; any disagreement was resolved by a consensus between reviewers.

### Data synthesis

The treatment effects for dichotomized outcomes were evaluated using the odds ratio (OR), and when studies adopted strategies to minimize confounding factors (e.g. adjustment propensity score/multivariable analyses), the adjusted odds ratio (aOR) was adopted; for continuous outcomes, the pooled mean difference (MD), or standardized mean difference (SMD) for different outcome measurements, was used. The variance was expressed with 95% confidence intervals (95% CI). When applicable, the outcome measures from the individual trials were combined through meta-analysis using random-effects models described by DerSimonian and Laird [18] because a certain degree of heterogeneity of population and treatments would be expected among interventions. Both crude and adjusted pooled treatment effects were reported in tables as well as in forest plots. A subgroup analysis for every included comparison was finally planned in order to better answer different questions (vs RT, v. RT + REBOA, vs no-REBOA). All tests were considered statistically significant, for *p* values less than 0.05. The analyses were performed by using RevMan Version 5.4 (Nordic Cochrane Center) [19].

### Quality of evidence

The quality of evidence of each outcome was judged through five dimensions (risk of bias, consistency of effect, imprecision, indirectness and publication bias) by the GRADE approach [20]. The evidence was downgraded from “high quality” by one level if serious, or by two levels if very serious limitations were found for each of the 5 dimensions. We presented a summary of findings describing the treatment effects, the quality of evidence and the reasons for limitations.

## Results

### Study selection

A total of 324 publications were selected for the analysis. No randomized controlled trials were found, one registered trial protocol not completed, five systematic reviews of observational studies and 10 observational studies met the eligibility criteria. A comparative evaluation between studies included in the systematic reviews and the primary studies resulting from the search strategy was performed: at the end of the search, 11 primary observational studies were included [21–31]. The flow diagram is reported in Supplement B.

### General characteristics

Overall, five studies assessed the comparison REBOA vs RT [21–24, 29], one study reported the comparison REBOA vs RT + REBOA [28] and five studies investigated the comparison REBOA vs no-REBOA [25–27, 30, 31]. Two of the selected studies were prospective [23, 24] and nine were retrospective [21, 22, 25–31]. The median injury severity score (ISS) across studies ranged from a minimum of 25 (IQR: 16–25) [25] and a maximum of 44 (IQR: 38–59) [28]. Blunt trauma was the most representative feature of patients across studies, except for one study that included only patients with penetrating trauma [25]. General characteristics are reported in Table 1.

**Table 1:** General characteristics

Study	Setting	Population	Intervention	Comparison	Outcome
Brenner 2018 [23] Prospective observational	Resuscitation in Trauma and Acute Care Surgery (AORTA) study was approved by the American Association for the Surgery	Adult trauma and acute care surgery (age ≥ 18) patients undergoing aortic occlusion (AO) in the acute phases after injury were enrolled Blunt trauma was common (58.6% of which 83% REBOA group and 48.5% RT) ISS: mean 38.2 (SD:18.9)	REBOA (*n* = 83) Unclear modality of intervention (full/partial)*	RT (*n* = 202)	In-hospital mortality, complication, units packed red blood cells, units fresh frozen plasma, health-related quality of life (neurologic outcomes: Glasgow Coma Outcomes Score)
Aso 2017 [22] Retrospective cohort study	Data from a national inpatient database in Japan	Trauma patients with uncontrolled haemorrhagic shock (*n* = 259); penetrating thoracic injuries were excluded Blunt trauma (100%) ISS: missing information	REBOA (n = 191) Unclear modality of intervention (full/partial)	RT (n = 68)	In-hospital mortality, ventilator-free days (VFDs), intensive care unit (ICU)-free days, total amount of fluid infusion within 1 day after admission (mL), total amount of transfusion within 1 day after admission (mL), total hospitalization costs
Abe 2016 [21] Retrospective cohort study	Japan Trauma Data Bank (JTDB) nationwide trauma registry	Trauma patients (*n* = 903) Blunt trauma was common (838/895; 93.6%) ISS: mean 34 (SD:25); mean 34 (SD:20)	REBOA (*n* = 636) Unclear modality of intervention (full/partial)	Resuscitative open aortic cross-clamping (RT) (*n* = 267)	In-hospital mortality, ED mortality, blood transfusion
DuBose 2016 [24] Prospective observational	Multicentre data from Trauma and Acute Care Surgery registry (8 American College of Surgeons level I centres)	Adult trauma and acute care surgery (age ≥ 18) patients undergoing aortic occlusion (AO) in the acute phases after injury (*n* = 114) Blunt trauma (62.3%) ISS: median 31.0(IQR: 30); median 31.5 (IQR: 22)	REBOA (*n* = 46) Unclear modality of intervention (full/partial)	AO (*n* = 68)	Haemodynamic stability, Improvement in haemodynamic red blood cell requirements, in-hospital mortality, ED mortality, Complications, health-related quality of life (neurologic outcomes: Glasgow Coma Outcomes Score)
Moore 2015 [29] Retrospective cohort study	Trauma registry from two Level 1 trauma Centres (Texas and Maryland-Baltimore)	Trauma patients in NCTH (*n* = 96) Blunt trauma (44.4% RT; 66.7% REBOA) ISS: median 34 (IQR:27–59); median 28 (IQR:17–43)	REBOA (*n* = 24) Unclear modality of intervention (full/partial)	RT (*n* = 72)	In-hospital mortality, ED mortality
Matsumara 2017* Retrospective cohort study	DIRECT-IABO Registry has been conducted by the Academic Committee in DIRECT in Japan	Trauma patients with refractory haemorrhagic shock Blunt trauma (96%) ISS: median 36 (IQR: 28–50); 44 (IQR: 38–59)	REBOA (*n* = 76) Partial occlusion (70% of participants) *	RT + REBOA group (*n* = 30)	In-hospital mortality
Nori 2015 [31] Retrospective cohort study	Japan Trauma Data Bank	Critically uncontrolled haemorrhagic shock limited to blunt trauma patients. Blunt trauma (100%) ISS: mean 32.4 (SD:16.4)	REBOA (*n* = 351) Unclear modality of intervention (full/partial)	Control group (*n* = 1456)	In-hospital mortality, health-related quality of life (neurologic outcomes: Glasgow Coma Outcomes Score)
García 2020 [25] Retrospective cohort study	Clinical records at Fundación Valle del Lili University hospital in Cali, Colombia level-I trauma centre from Colombia	Patients with torso trauma who underwent surgical intervention for haemorrhage control excluded blunt trauma. Penetrating trauma (100%) ISS: median 25 (IQR: 16–25)	REBOA (*n* = 28) Partial occlusion*	Control group (*n* = 317)	In-hospital mortality, PRBCs A in first 6 h , Plasma A in first 6 h, platelet A in first 6 h, Cryo A in first 6 h , Crystalloids in first 24 h, Thoracic damage control, Abdominal damage control, complications
Inoue 2016 [26] Retrospective cohort study	Japan Trauma Data Bank	Patients with severe torso trauma Blunt trauma (93.8%) ISS: median 35 (IQR: 25–50); median 36 (IQR: 25–50)	REBOA (*n* = 625) Unclear modality of intervention*	Control group (*n* = 625)	In-hospital mortality, ED mortality
Joseph 2019 [27] Retrospective case-control study	ACSTQIP database and identified all patients who received REBOA within 1 h of admission	Trauma patients after REBOA placement Blunt trauma (95%) ISS: median 28 (IQR:17–35); median 29 (IQR: 18–38)	REBOA (*n* = 140) Unclear modality of intervention*	Control group (*n* = 280)	In-hospital mortality, ED mortality, transfusion requirements at 4 h and 24 h after injury, in-hospital complications (deep venous thrombosis, pulmonary embolism, stroke, myocardial infarction, extremity compartment syndrome, health-related quality of life (neurologic outcomes: Glasgow Coma Outcomes Score)
Yamamoto 2019 [30] Retrospective cohort study	Japan Trauma Data Bank	Severely injured patients Blunt trauma (96% REBOA; 94% controls) ISS: mean 35 (SD: 13); 33 (SD: 11)	REBOA (*n* = 117)	Control group (*n* = 117)	Survival at 28 days, a composite of in-hospital death, transfusion in number of patients

Legend: *AO* open aortic occlusion, *ACC* resuscitative open aortic cross-clamping, *BMI* body mass index, *JCS* Japan Coma Scale, *ED* emergency department, *NCTH* non-compressible torso haemorrhage, *RTS* revised trauma score, *RT* resuscitative thoracotomy with aortic cross-clamping, *TMPM-ICD9* the Trauma Mortality Prediction Model based on the ICD 9th Revision, TRISS Trauma and Injury Severity Score

### Overall mortality

All studies (*n* = 11) reported overall mortality data (Table 2). Most of these (*n* = 9) did not report the time from injury to death, similarly for the outcome at discharge without a specific time frame. In addition, five studies evaluated overall mortality in the emergency department, while four studies assessed the mortality at 24 h and three studies reported data at 1 month (Supplement C).

**Table 2:** Overall in-hospital mortality. Data are collected for the last available observation when time of follow up is specified

Overall mortality	REBOA			Control			Time/setting	OR adjusted/matched	Description of adjustment
	***N***	Tot	%	***n***	Tot	%			
Aso 2017 [22] (REBOA vs RT)	90	191	47	48	68	70.6	Time frame not reported	Hazard ratio = 0.94; 95%CI = 0.60–1.48^§^ OR 0.821; 95% CI 0.306–1.234	Adjusted propensity score
Brenner 2018 [23] (REBOA vs RT)	75	83	90.3	197	202	97.5	24 h	OR = 0.24; 95% CI 0.08–0.75	None
Abe 2016 [21] (REBOA vs RT)	405	636	63.7	210	267	78.7	Time frame not reported ED	OR 0.261 95%CI 0.130–0.523 Pair-matched *n* = 304	Adjusted propensity score
DuBose 2016 [24] (REBOA vs RT)	33	46	71.7	57	68	83.8	ED 24 h	OR = 0.263; 95% CI = 0.043–1.609	not reported (regression)
Moore 2015 [29] (REBOA vs RT)	15	24	62.5	65	72	90.3	time frame not reported ED	None	None
Matsumara 2017 (REBOA vs REBOA+RT)	41	76	53.9	27	30	90.0	24 h 1 month At discharge	None	None
Nori 2015* [31] (REBOA vs no-REBOA)	259	351	73.8	709	1456	48.7	Time frame not reported	OR = 2.97; 95% CI = 2.29–3.84 Pairs matched 1:5	Adjusted propensity score
García 2020 [25] (REBOA vs no-REBOA)	5	28	17.8	48	317	15.1	Time frame not reported	OR = 0.20; 95%CI 0.05–0.77	Adjusted propensity score
Inoue 2016* [26] (REBOA vs no-REBOA)	386	625	61.7	283	625	45.3	Time frame not reported ED	OR = 1.95, 95% CI 1.56–2.45	Adjusted propensity score °
Joseph 2019* [27] (REBOA vs no-REBOA)	50	140	35.7	53	280	18.9	ED overall	OR= 2.38; 95% CI= 1.51–3.76	Adjusted propensity score
Yamamoto 2019* [30] (REBOA vs no-REBOA)	64	117	54.7	79	117	67.5	Time frame not reported	OR = 0.58; 95% CI = 0.34–0.99	Adjusted propensity score

^§^To be able to pool the adjusted odds ratios in a meta-analysis, the hazard ratio reported in the study by Aso et al. 2017 [22] was converted to an odds ratio. For the procedure, we assumed that the hazard ratio is a type of relative risk and, thus, is asymptotically similar to a relative risk. Then, using the inverse probability weighted binomial model we transformed the adjusted hazard ratio of mortality reported in the study by Aso to an odd ratio. Following this approach, we obtained an adjusted odds ratio of mortality (Aso: OR 0.821; 95% CI 0.306–1.234)

*Data were reported only for pairs

°Mortality was estimated via linear regression analysis, and time variables were estimated via bootstrapping

**Fig. 1:**
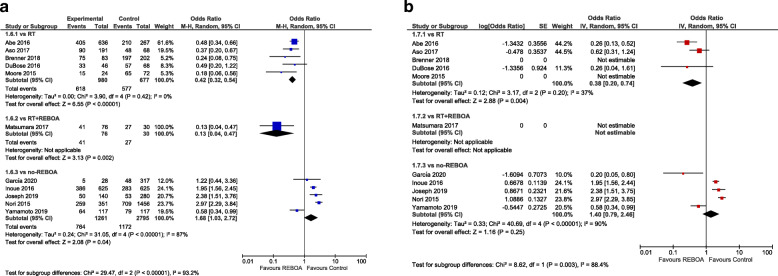
**a** Overall crude odds ratios for in-hospital mortality (REBOA vs control – subgroups: vs RT; vs RT with REBOA; vs no-REBOA). **b** Overall adjusted odds ratios for in-hospital mortality (REBOA vs control — subgroups: vs RT; vs RT with REBOA; vs no-REBOA)

**Fig. 2:**
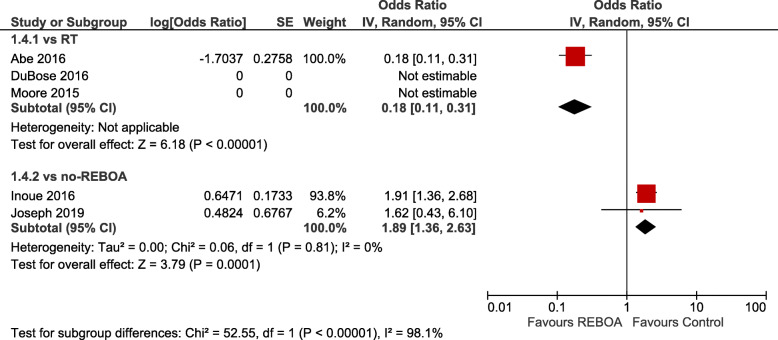
Adjusted odds ratios for mortality in ED (REBOA vs control — subgroups: vs RT; vs no-REBOA)

**Fig. 3:**
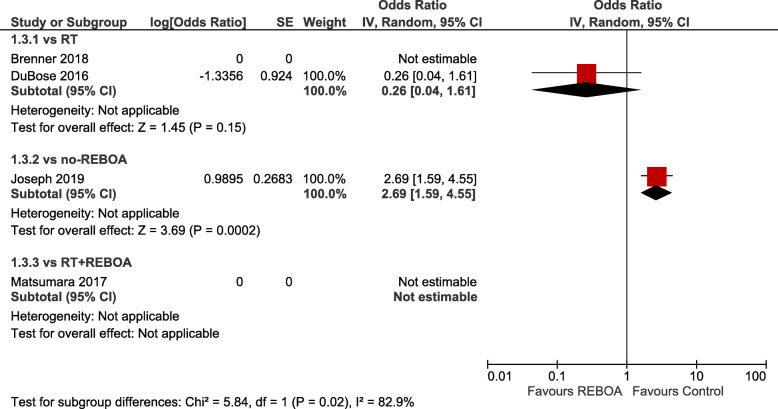
Adjusted odds ratios for mortality at 24 h (REBOA vs control — subgroups: vs RT; vs RT with REBOA; vs no-REBOA)

Crude summary estimates found statistically significant differences in favour of REBOA when compared to RT (OR 0.42; 95% CI 0.32–0.54, *I*^2^ = 0) or RT with REBOA (OR 0.13; 95% CI 0.04–0.47) and against REBOA when compared to no-REBOA (OR 0.68; 95% CI 1.03–2.72, *I*^2^ = 87). Adjusted summary estimates confirmed a statistically significant difference favouring REBOA vs. RT (aOR 0.38; 95% CI 0.20–0.74, *I*^2^ = 37), whereas no significant difference was present when REBOA was compared to no-REBOA (aOR 1.40; 95% CI 0.79–2.46, *I*^2^ = 90). Figure 1 a shows the crude pooled treatment effects, whereas Fig. 1 b shows the adjusted pooled treatment effects of overall mortality of the studies that provided data at discharge or at the last available follow-up adjusted by matching with the propensity score or by regression. Figure 2 shows mortality in the emergency department, while Fig. 3 presents 24-h mortality. Individual studies investigated mortality at 1 month for RT vs REBOA [28] (aOR not estimable) and REBOA vs. no-REBOA (aOR 0.77, 95% CI 0.36–1.61) [27, 30], without evidence of significant effects (Supplement C).

### Volume of infused blood components

Six studies investigated the volume of blood components. Some studies demonstrated a statistically significant decrease in the number of plasma [24, 25] and platelets [27] infused to the patients and in the number of patients who needed transfusion [30]. However, heterogeneity in the reporting of outcome measurements prevents a quantitative analysis and data are reported in Table 3.

**Table 3:** Volume of blood components

Autore	Outcome	Units	REBOA	Control^**a**^	***p*** value
**Cryoprecipitate**					
Dubose 2016 [24]	Cryoprecipitate 24 h	Median (IQR)	1 (11)	0 (1)	0.14
Garcia 2020 [25]	Cryoprecipitate 6 h	Median (IQR)	6.5 (0–10)	0 (0–0)	0.21
**Crystalloids**					
Garcia 2020 [25]	Crystalloids 24 h millilitres	Median (IQR)	4649 (3290–6329)	4420 (2705–6350)	0.13
Dubose 2016 [24]	Crystalloids 24 h litres	Median (IQR)	4 (5)	3 (5)	0.12
**Plasma**					
Joseph 2019 [27]	Plasma 24 h	Median (IQR)	9 (6–20)	10 (7–20)	0.17
Brenner 2018 [23]	Plasma 24 h	Median (IQR)	9 (16)	4 (9)	0.11
DuBose 2016 [24]	Plasma 24 h	Median (IQR)	14.5 (18)	6 (18)	< 0.001
Joseph 2019 [27]	Plasma 4 h	Median (IQR)	3 (2–5)	3 (2–6)	0.001
Garcia 2020 [25]	Plasma 6 h	Median (IQR)	4 (2.5–6)	0 (0–4)	< 0.001
**Platelets**					
Joseph 2019 [27]	Platelets 24 h	Median (IQR)	7 (3–13)	8 (3–12)	< 0.001
DuBose 2016 [24]	Platelets 24 h	Median (IQR)	5.5 (12)	1.5 (11)	0.5
Joseph 2019 [27]	Platelets 4 h	Median (IQR)	4 (3–9)	4 (3–8)	0.05
Garcia 2020 [25]	Platelets 6 h	Median (IQR)	0.5 (0–6)	0 (0–0)	0.05
**PRBCs**					
Joseph 2019 [27]	PRBCs 24 h	Median (IQR)	9 (5–20)	10 (4–21)	0.3988
Brenner 2018 [23]	PRBCs 24 h	Median (IQR)	10 (21)	7.8 (10)	0.654
DuBose 2016 [24]	PRBCs 24 h	Median (IQR)	20.5 (18)	13.5 (18)	0.343
Joseph 2019 [27]	PRBCs 4 h	Median (IQR)	6 (3–8)	7 (3–9)	0.872
Garcia 2020 [25]	PRBCs 6 h	Median (IQR)	5 (3–9)	2 (0–4)	0.149
**Total amount of transfusion**					
Aso 2016 [22]	Total amount of transfusion within 1 d after admission: average (SD), mL	media (sd)	2.396 (1.872)	2.820 (2.782)	0.697
**Transfusion in number of patients**					
Abe 2016 [21]	Transfusion in number of patients	*n* (%)	542 (85%)	197 (74%)	0.001
Yamamoto 2019 [30]	Transfusion in number of patients	*n* (%)	111 (95%)	113 (97%)	< 0.001

^a^RT (Abe 2016, Aso 2016, DuBose 2016, Brenner 2018), RT + REBOA (Matsumara 2017), non-REBOA (Yamamoto 2019, Joseph 2019, Garcia 2020)

### Health-related quality of life

The Discharge Glasgow Coma Scale (Discharge GCS) among survivors, as a proxy of health-related quality of life outcome, was reported in four studies [23, 24, 27, 31].

A study [24] found no significant differences between the 2 groups (median REBOA group, 15 points; median RT group, 15 points, *p* = 0.766) reported, and in another study [23], the Discharge GCS was in favour of the REBOA group, but only in the pre-hospital cohort (median REBOA group, 9 points; median RT group, 3 points, *p* = 0.026).

In the last two studies [27, 31] only a subgroup analysis in patients who received REBOA was performed. Surviving patients had higher Glasgow Coma Scale (GCS) than non-survivors in both Norii et al., 2015 (mean GCS, 11.6 survivors vs. 7.2 non-survivors, *p* = 0.0001) and Joseph et al. 2019 studies (median (IQR) GCS, 15 (13-15) survivors vs 3 (3-13) non-survivors, *p* = 0.04).

### Adverse effects

Adverse events for both groups of treatment (REBOA vs. RT) were reported in three studies [23, 24, 27], whereas one study [25] reported adverse events only for the REBOA group. Overall, the most frequently reported complications from the studies were amputation, haematoma and pseudo-aneurysm, shown in Supplement C.

### Other outcomes

Two studies reported the number of subjects in which the technique was successfully performed (> 91%) [23, 24]. Two studies reported the temporary time to control haemorrhage [27, 28]. One study [28], investigating REBOA vs REBOA +RT, reported the control time for bleeding from arrival at the scene. Patients with arterial access achieved within 21.5 min of arrival demonstrated immediate subsequent haemostasis. Heterogeneous measurements for the improvement in haemodynamics (blood pressure and heart rate) are reported in two studies [23, 24]. Supplement C descriptively reported data.

### Internal validity and quality of evidence

Seven studies were judged to be of good quality and four of fair quality **(**Supplement D). Certainty of evidence ranged from very low to low with no serious risk of bias. We downgraded the evidence for serious indirectness and imprecision of the estimates (Supplement E).

### Algorithm for decision-making

**Fig. 4:**
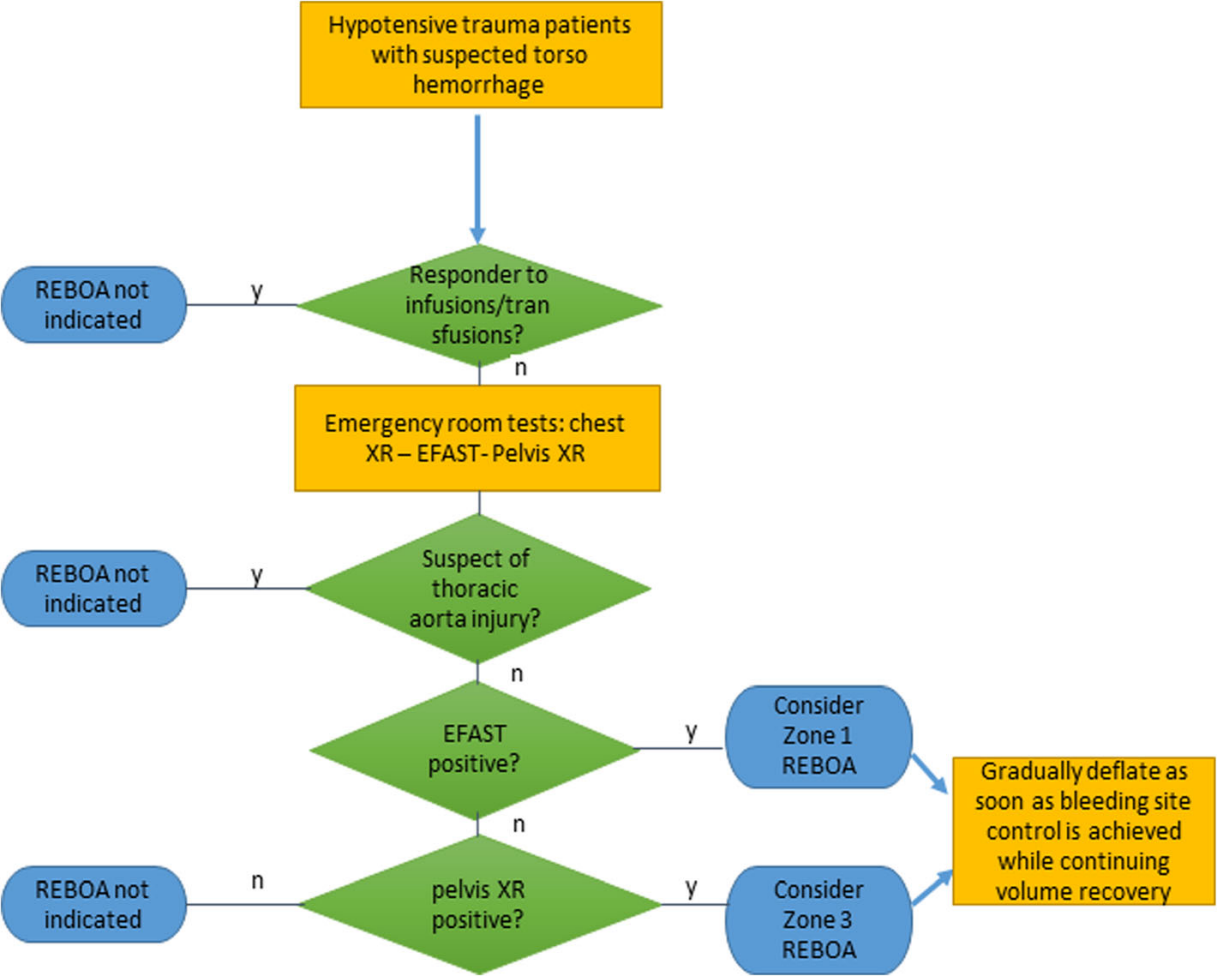
An algorithm for REBOA indications

REBOA might be considered in haemodynamically unstable patients, unresponsive to initial resuscitation for a suspected torso haemorrhage, as indicated by E-FAST positive for free peritoneal fluid and/or pelvis x-ray indicating fracture of the ring. REBOA is inflated in zone 1 if positive E-FAST or impending cardiac arrest and zone 3 if pelvic fracture. REBOA is progressively deflated as soon as the bleeding site is controlled with temporary or definitive surgical techniques, while continuing volume replacement. REBOA is not indicated in the suspicion of injury of the thoracic aorta and if emergency room diagnostic tools fail to demonstrate a torso haemorrhage. Figure 4 describes an algorithm for REBOA indications.

## Discussion

To our knowledge, this is the first comprehensive systematic review and meta-analysis assessing all comparisons between REBOA and RT and REBOA versus no-REBOA, considering many critical and important outcomes. With low quality of evidence, adjusted overall estimates found a difference in favour of REBOA when compared to RT (aOR 0.38; 95% CI 0.20–0.74). With very low quality of evidence, REBOA when compared to no-REBOA (aOR 1.40; 95% CI 0.79–2.46) did not show a significant difference in outcomes. Adverse events were poorly reported across studies: only four studies reported complications such as amputation, haematoma and pseudoaneurysm.

Our literature search found five systematic reviews about the use of REBOA. However, two investigated the REBOA in a variety of clinical settings [10, 32], and one focussed only on adverse events revising case series studies [33]. Our results are consistent with other two systematic reviews [34, 35] where the comparison of REBOA versus RT found similar quantitative findings (aOR 0.42; 95%CI 0.17–1.03; OR 0.25; 95%CI 0.11–0.56, respectively). Nevertheless, our review updated the evidence including the last three years of publication.

All the studies where REBOA was compared to RT [21–24, 29] demonstrated a clear survival benefit in very sick patients. This statistical significance can be biased since the most serious patients in cardiac arrest or imminent cardiac arrest undergo a RT, while more stable patients might be considered for REBOA. In very sick patients with very low critical tissue and organ perfusion or impending cardiac arrest, extreme resuscitative manoeuvres are required as a bridge to save time to definitive bleeding control or other potential reversible injury management. RT is a maximally invasive procedure in the trauma setting with a survival of less than 10% but associated with a negative perception because often used as the last resort in patients beyond saving [36, 37]. In this comparison, patient selection separates patients into the dead-or-nearly-dead (RT) and the alive-but-severe-shock (REBOA) with no valid conclusions regarding the superiority or preference of REBOA over RT. Therefore, in the absence of randomized trials, REBOA can only be considered an option for the clinician in very sick haemorrhagic patients following trauma. The more valuable contribution to the literature should come from the comparison of REBOA and no-REBOA. However, when REBOA has been compared with no-REBOA in our analyses [25–27, 30] a clinical benefit was not observed. The available published studies range from very low and low certainty of evidence, including patients with brain injuries or chest injuries on which REBOA has no benefit and sometimes can be harmful. Moreover, the evidence was limited to observational studies. In all studies, the clinical indication to REBOA was haemorrhage from a pelvic fracture or abdominal injury, conditions that recognize optimal clinical results with standard damage control approaches, such as abdominal or extraperitoneal packing [38]*.* Both manoeuvres improve haemodynamics and survival and are easily performed by surgical personnel with specific experience.

REBOA stops flow totally or partially below the occlusion level (zone I or III), inflation limits the bleeding but also does produce ischemia both regionally and systemically. Given the tendency towards reperfusion injury, REBOA has a limited time window of application before the complications overcome the benefit of intervention [39, 40]. REBOA is likely not clinically better than the standard and more consolidated damage control interventions for bleeding control in non-compressible torso haemorrhagic shock, but can be implemented as a life-saving haemostatic bridge, if damage control surgery is not immediately available after prompt evaluation and indications based on clinical characteristics of patients. Furthermore, many variables can affect survival in this category of patients: pre-hospital time, prompt recognition of bleeding site(s), availability of expert surgeon, time to operating room, appropriate transfusion protocol, physiology of the patient. Time to operating room and physiologic state of patients may predict outcome as importantly as does whether a REBOA is used [41]. Due to these considerations, further investigations with an adequate volume of cases considering all possible confounders can help to understand the efficacy of REBOA in torso haemorrhages.

## Limitations

Our review is the most comprehensive effort in the management of haemorrhage in major trauma patients; however, several limitations must be addressed. Although our quantitative synthesis shows that REBOA is associated with lower mortality when compared to RT, these results could be flawed by the presence of patient selection for indication bias and survival bias within the individual observational studies [42]. Indication bias arises when patients are classified on the basis of the non-randomized intervention they received during the natural course of their medical treatment. Survival bias appears when comparing groups in which patients may die before treatment is initiated [43]. Clinical conditions (e.g. cardiac arrest) strongly influenced the treatment indication and so the assignment of patients in the RT or no-REBOA group. In the emergency department, RT is performed in patients who are experiencing post-traumatic cardiac arrest, while REBOA is indicated for trauma patients who are in an uncontrolled haemorrhagic shock for a pelvic fracture or abdominal fluid detected on an initial ultrasonography scan in the trauma bay [27]. For these reasons, some studies may have an inadequate control group (i.e. patients who did not undergo REBOA placement and/or RT). We have overcome this limitation by subgrouping the patients, who underwent thoracotomy in the ED, patients who underwent REBOA and those who did not undergo REBOA. Unfortunately, we did not find RCTs, the most reliable evidence on the effectiveness of interventions [44] which minimize the risk of bias and confounding factors influencing the results [45]. Even if performing RCTs can be unethical in life-threatening situations, challenging to design and deliver it is not impossible: a recent mapping review has highlighted that evidence from trials in prehospital trauma is sparse and can be prioritized [46]. We call for the need for further randomized trials of REBOA vs RT and REBOA vs no-REBOA in order to assure well-matched patients.

The use of REBOA should take into account skills, high expertise on their applicability [47, 48], acceptability of clinicians and cost [49, 50]. For optimal success, REBOA requires careful system-wide multidisciplinary implementation [51]. Institutions are responsible for analysing qualifications for providers to perform REBOA [42] as well as evaluating system capabilities [52]. A very small number of trauma centres have an extensive experience with REBOA; thus, these results may not be generalizable to all trauma centres [42]. Finally, we included studies with a heterogeneous use of REBOA which should be taken into account (catheter size, occlusion zone, protocols, physiologic indications for REBOA insertion).

## Conclusion

Among non-compressible torso injuries, we found a positive effect on overall mortality of REBOA when compared to RT but no valid conclusions can be made due to selection bias, while not statistically significant the comparison of REBOA versus no-REBOA from which the most valuable contribution for clinical practice is drawn. REBOA should be promoted in specific training programs in an experimental setting in order to test its effectiveness as temporary management to haemorrhage control and resuscitation. Prospectively assessed data, with specific inclusion and exclusion criteria, ideally in a randomized controlled trial, should be planned in order to limit the bias coming from observational studies. Future studies must address specific indications for REBOA to know which population could benefit the most from its use.

## Supplementary Information

**Additional file 1.**

## Data Availability

All data generated or analysed during this study are included in this published article [and its additional files]. Row data are stored in an open platform at the following link: https://osf.io/ntxvj/

## Abbreviations

aOR: Adjusted odds ratio
GCS: Glasgow Coma Scale
IQR: Interquartile range
ISS: Injury severity score
MD: Mean difference
MOOSE: Meta-Analysis of Observational Studies in Epidemiology
OR: Odds ratio
PRISMA: Preferred Reporting Items for Systematic Reviews and Meta-Analyses
RCT: Randomized controlled trials
REBOA: Resuscitative Endovascular Balloon Occlusion of the Aorta
RoB: Cochrane Risk of Bias
RT: Resuscitative thoracotomy
SMD: Standardized mean difference
SNLG: Sistema Nazionale Linee Guida
