# Supplementary material for: Resuscitative endovascular balloon occlusion of the aorta (REBOA) in patients with major trauma and uncontrolled haemorrhagic shock: a systematic review with meta-analysis
Source: World J Emerg Surg. 2021 Aug 12;16:41. doi: 10.1186/s13017-021-00386-9 (PMC8358549; doi:10.1186/s13017-021-00386-9)
Supplement: Supplementary file 1 — Additional file 1. [file 13017_2021_386_MOESM1_ESM.docx]

**Resuscitative Endovascular Balloon Occlusion of the Aorta (REBOA) in patients with major trauma and uncontrolled haemorrhagic shock: a systematic review with meta-analysis**

[**Supplement A**. Question and Search strategy 2](#_Toc63956854)

[**Supplement B.** Flow Diagram of study selection PRISMA 6](#_Toc63956855)

[**Supplement C**. Additional analyses 7](#_Toc63956856)

[**Mortality in Emergency Department** 7](#_Toc63956857)

[**Mortality at 24 h** 7](#_Toc63956858)

[**Mortality at 1 month** 8](#_Toc63956859)

[**Mortality at 12 months** 8](#_Toc63956860)

[**Adverse effects** 9](#_Toc63956861)

[**Temporary time to control hameorrage** 9](#_Toc63956862)

[**Improvement in haemodynamic (blood pressure and heart rate)** 10](#_Toc63956863)

[**Failure/success of REBOA technique** 10](#_Toc63956864)

[**Supplement D**. Internal validity 11](#_Toc63956865)

[**Supplement E**. Summary of Findings Tables 13](#_Toc63956866)

# **Supplement A**. Question and Search strategy

**Medline search**

| 1. | (trauma* or polytrauma*).ti,ab. |
| --- | --- |
| 2. | ((serious* or severe* or major or life threaten*) adj3 (accident* or injur* or fall*)).ti,ab. |
| 3. | multiple trauma/ |
| 4. | wounds, gunshot/ or wounds, stab/ or accidents, traffic/ or accidental falls/ or blast injuries/ or accidents, aviation/ |
| 5. | ((motor* or motorbike* or vehicle* or road or traffic or car or cars or cycling or bicycle* or automobile* or bike* or head on or pile up) adj3 (accident* or crash* or collision* or smash*)). ti,ab. |
| 6. | (mvas or mva or rtas or rta). ti,ab. |
| 7. | (stabbed or stabbing or stab or gunshot* or gun or gunfire or firearm$ or bullet* or knife* or knives or dagger). ti,ab. |
| 8. | or/1-7 |

**Embase search terms**

| 1. | (trauma* or polytrauma*). ti,ab. |
| --- | --- |
| 2. | ((serious* or severe* or major or life threaten*) adj3 (accident* or injur* or fall*)).ti,ab. |
| 3. | multiple trauma/ |
| 4. | gunshot injury/ or stab wound/ or traffic accident/ or falling/ or blast injury/ or aircraft accident/ |
| 5. | ((motor* or motorbike* or vehicle* or road or traffic or car or cars or cycling or bicycle* or automobile* or bike* or head on or pile up) adj3 (accident* or crash* or collision* or smash*)).ti,ab. |
| 6. | (mvas or mva or rtas or rta).ti,ab. |
| 7. | (stabbed or stabbing or stab or gunshot* or gun or gunfire or firearm$ or bullet* or knife* or knives or dagger).ti,ab. |
| 8. | or/1-7 |

**Cochrane search terms**

| 1. | MeSH descriptor: [multiple trauma] this term only |
| --- | --- |
| 2. | (trauma* or polytrauma*):ti |
| 3. | ((serious* or severe* or major) near/3 (accident* or injur* or fall*)):ti |
| 4. | MeSH descriptor: [wounds, gunshot] this term only |
| 5. | MeSH descriptor: [wounds, stab] this term only |
| 6. | MeSH descriptor: [accidents, traffic] this term only |
| 7. | MeSH descriptor: [accidental falls] this term only |
| 8. | MeSH descriptor: [blast injuries] this term only |
| 9. | MeSH descriptor: [accidents, aviation] this term only |
| 10. | ((motor* or motorbike* or vehicle* or road or traffic or car or cars or cycling or bicycle* or automobile* or bike*) near/3 (accident* or crash* or collision* or smash*)):ti |
| 11. | (mvas or mva or rtas or rta):ti |
| 12. | (stabbed or stabbing or stab or gunshot or gun or gunfire or firearm* or bullet or knife* or knives or dagger or shot):ti |
| 13. | {or #1-#12} |

**CRD search terms**

| 1. | MeSH descriptor multiple trauma |
| --- | --- |
| 2. | MeSH descriptor wounds, gunshot |
| 3. | MeSH descriptor wounds, stab |
| 4. | MeSH descriptor accidents, traffic |
| 5. | MeSH descriptor accidental falls |
| 6. | MeSH descriptor blast injuries |
| 7. | MeSH descriptor accidents, aviation |
| 8. | ((trauma* or polytrauma*)) |
| 9. | (((serious* or severe* or major or life threaten*) near3 (accident* or injur* or fall*))) |
| 10. | (((mvas or mva or rtas or rta))) |
| 11. | (((stabbed or stabbing or stab or gunshot* or gun or gunfire or firearm* or bullet* or knife* or knives or dagger))) |
| 12. | ((((motor* or motorbike* or vehicle* or road or traffic or car or cars or cycling or bicycle* or automobile* or bike*) near3 (accident* or crash* or collision* or smash*)))) |
| 13. | (#1 or #2 or #3 or #4 or #5 or #6 or #7 or #8 or #9 or #10 or #11 or #12) |

##### Haemorrhagic shock / population

| 1. | Shock, hemorrhagic/ct |
| --- | --- |
| 2. | Hemorrhagic shock/ct |
| 3. | Hemorrhagic(a) shock/ti,ab |
| 4. | Haemorrhagic(a) shock/ti,ab |
| 5. | Traumat?(a)shock/ti,ab |
| 6. | Hemorrhage(l)prevention & control/ct |
| 7. | Hemorrhage+nt/ct |
| 8. | Hemorrhage#/ti,ab or haemorrhage#/ti,ab or bleed?/ti,ab |
| 9. | Damage# control#/ti,ab |
| 10. | Exsanguination/ct or exsanguinat?/ti,ab |
| 11. | Shock+nt/ct |
| 12. | Hypovolemia/ct or hypovol!em?/ti,ab |
| 13. | Hypoperfus?/ti,ab |
| 14. | Bloodloss/ti,ab or blood(3w)loss/ti,ab |
| 15. | Olig!em?/ti,ab |

**INTERVENTION**

| 1. | Resuscitation+nt/ct and balloon occlusion/bi and aorta+nt/ct |
| --- | --- |
| 2. | Resuscitat?/ti,ab and balloon#/ti,ab and occlusi?/ti,ab and |
| 3. | Aorta/ti,ab |
| 4. | Resuscitat?/ti,ab and endovascular/ti,ab and balloon#/ti,ab |
| 5. | And OCCLUSI?/ti,ab |
| 6. | Open repair/ti,ab |
| 7. | Reboa/ti,ab |
| 8. | Angioplasty balloon#/bi and aorta/bi |
| 9. | Aort? And balloon# and occlusi?/ti,ab |
| 10. | Aort? And balloon# and tampona?/ti,ab |

##### Excluded study designs and publication types

The following study designs and publication types were removed from retrieved results using **the NOT** operator.

**Medline search terms**

| 1. | letter/ |
| --- | --- |
| 2. | editorial/ |
| 3. | news/ |
| 4. | exp historical article/ |
| 5. | anecdotes as topic/ |
| 6. | comment/ |
| 7. | case report/ |
| 8. | (letter or comment*).ti. |
| 9 | animals/ not humans/ |
| 10. | exp animals, laboratory/ |
| 11 | exp animal experimentation/ |
| 12 | exp models, animal/ |
| 13 | exp rodentia/ |
| 14 | (rat or rats or mouse or mice).ti. |
| 15 | or/1-14 |

**Embase search terms**

| 1. | letter.pt. or letter/ |
| --- | --- |
| 2. | note.pt. |
| 3. | editorial.pt. |
| 4. | case report/ or case study/ |
| 5. | (letter or comment*).ti. |
| 6. | animal/ not human/ |
| 7. | nonhuman/ |
| 8. | exp animal experiment/ |
| 9. | exp experimental animal/ |
| 10. | animal model/ |
| 11. | exp rodent/ |
| 12. | (rat or rats or mouse or mice).ti. |
| 13. | or/1-12 |

# **Supplement B.** Flow Diagram of study selection PRISMA

COCHRANE
(n =10)

EMBASE and MEDLINE
(n =315)

Records after duplicates removed
(n =324)

Eligibility

Screening

Full-text articles excluded, with reasons
(n = 81):

n= 1 wrong population

n= 1 wrong intervention

n= 52 wrong study design

n= 7 out of scope

n = 11 abstract/conference proceedings

n = 3 duplicate

n =4 no outcome of interest

n = 2 awaiting assessment (language)

Records screened
(n =324)

Full-text articles assessed for eligibility
(n =96)

Studies included in quantitative synthesis
(n = 11 observational studies)

total studies included in qualitative synthesis
(n = 11 observational studies)

Studies included (n = 5 systematic reviews and meta- analysis; n = 10 observational)

Records excluded
(n =228)

Identification

Included

# **Supplement C**. Additional analyses

## **Mortality in Emergency Department**

**Table 1.** Mortality in Emergency Department

|  | **REBOA** | | **Control *** | | **Adjusted estimate** | **Description of adjustment** |
| --- | --- | --- | --- | --- | --- | --- |
|  | **n** | **tot** | **n** | **tot** |  |  |
| Abe 2016 | 137 | 636 | 130 | 267 | OR 0.182 95%CI 0.106-0.31  n=299/304 | Propensity score |
| DuBose 2016 | 25 | 46 | 31 | 68 | none | none |
| Moore 2015 | 4 | 24 | 45 | 72 | none | none |
| Inoue 2016 | 107 | 625 | 61 | 625 | OR 1.91, 95% CI 1.36 – 2.67 | Propensity score§ |
| Joseph 2019 | 4 | 140 | 5 | 280 | OR 1.62 95% CI 0.43 – 6.12 | Adjusted Propensity score |

§ Mortality was estimated via linear regression analysis, and time variables were estimated via bootstrapping.

*** RT (**Abe 2016, DuBose 2016, Moore 2015), **non-REBOA** (Inoue 2016, Joseph 2019).

## **Mortality at 24 h**

**Table 2.** Mortality at 24 h

|  | **REBOA** | | **Control*** | | **Adjustment** | **Description of adjustement** |
| --- | --- | --- | --- | --- | --- | --- |
|  | **n** | **tot** | **n** | **tot** |  |  |
| Joseph 2019 | 37 | 140 | 33 | 280 | OR 2.69, 95% CI 1.59 – 4.53 | Adjusted Propensity score |
| Matsumara 2017 | 30 | 76 | 24 | 30 | none | none |
| Dubose 2016 | 33 | 46 | 57 | 68 | OR= 0.263; 95% CI= 0.043 – 1.609 | Adjusted (regression) |
| Brenner 2018 | 75 | 83 | 197 | 202 | none | none |

***RT (**DuBose 2016, Brenner 2018), **RT+REBOA (**Matsumara 2017), **non-REBOA (**Joseph 2019).

## **Mortality at 1 month**

**Table 3.** Mortality at 1 month

| **Overall mortality at discharge** | **REBOA** | | **Control*** | | **Adjustment** | **Description of adjustement** |
| --- | --- | --- | --- | --- | --- | --- |
|  | **n** | **tot** | **n** | **tot** |  |  |
| Yamamoto 2019 | 62 | 117 | 78 | 117 | OR 0.56, 95% CI 0.33 – 0.96 | Adjusted Propensity score |
| Matsumara 2017 | 37 | 76 | 27 | 30 | none |  |
| Joseph 2019 § | 9 | 140 | 15 | 280 | OR 1.21, 95%CI 0.52 – 2.85 | Adjusted Propensity score |

§ after 24 h

***** **RT+REBOA (**Matsumara 2017), **non-REBOA (**Yamamoto 2019, Joseph 2019)

**
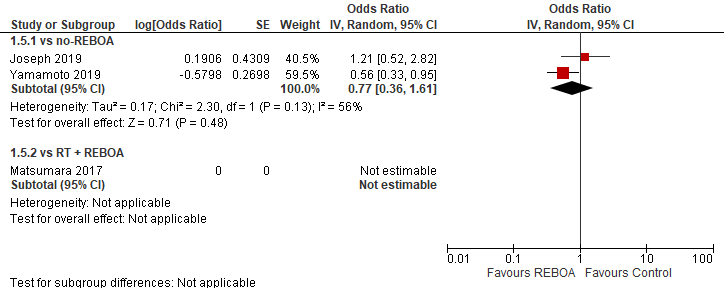
**

**Figure 1.** Adjusted odds ratios for mortality at 1 month

## **Mortality at 12 months**

none of the included studies reported this outcome.

## **Adverse effects**

**Table 4.** The most reported adverse events

|  | *REBOA* | *Control* | *p-value* |
| --- | --- | --- | --- |
| *Need of amputation* | | |  |
| Brenner 2018 | *1 (1.2%)* | *-* |  |
| DuBose 2016 | *0 (0%)* | *-* |  |
| Joseph 2019 | *5 (3.6%)* | *2 (0.7%)* | *0.04* |
| *Hematoma* | | |  |
| Brenner 2018 | *0 (0%)* | *-* |  |
| DuBose 2016 | *0 (0%)* | *-* |  |
| *Pseudoaneurism* | | |  |
| Brenner 2018 | *0 (0%)* |  |  |
| DuBose 2016 | *1 (2.2%)* |  |  |

## **Temporary time to control haemorrhage**

**Table 5**. Time to control haemorrhage in Joseph 2019

| **Haemorrhage control intervention** | **No-REBOA (n=280)** | **REBOA (n=140)** | **p-value** |
| --- | --- | --- | --- |
| Angioembolization | 85 (30.4) | 40 (28.6) | 0.18 |
| Time to angioembolization median (IQR), min | 46(31-39) | 59 (39-78) | 0.04 |
| Laparotomy | 190 (67.9) | 96 (68.6) | 0.33 |
| Time to laparotomy, median IQR | 33 (26-62) | 45 (35-69) | 0.04 |

**Table 6.**Time to control haemorrhage in Matsumara 2017

|  | REBOA alone (n=76) | REBOA+RT (n=30) | p |
| --- | --- | --- | --- |
| Time course (min) |  |  |  |
| Arrival to REBOA | 60 (27-85) | 32 (20-63) | 0.026 |
| Arrival to definitive care | 71 (50-101) | 25(11-60) | <0.001 |

Legend: The data are presented as median (25th–75th percentile)

“The shorter arrival to access time and lower ISS were significantly associated with increased survival in hemorrhagic patients undergoing REBOA. Patients with arterial access obtained within 21.5 minutes from arrival demonstrated prompt subsequent hemostasis and better survival curves. Proactive early access in the resuscitation phase may be associated with survival outcomes.”

## **Improvement in haemodynamic (blood pressure and heart rate)**

**Table 7.** Improvement in haemodynamic

|  | **REBOA (n=46)** | **RT (n=68)** | **P value** |
| --- | --- | --- | --- |
| ***Dubose 2016*** |  |  |  |
| Improvement in hemodynamics with aortic occlusion, n (%) | 29(67.4%) | 42(61.8%) | 0.544 |
| Hemodynamic stability (SBP consistently above 90 mm Hg) achieved with aortic occlusion, n (%) | 22 (51.2%) | 19(27.9%) | 0.014 |
| Hemodynamic improved with 2^nd^aortic occlusion, n (%) | 2/2 (100%) | 7/9 (77.8%) | 0.244 |
| Hemodynamic stability (SBP consistently > 90 mm Hg) achieved with 2^nd^ aortic occlusion, n (%) | 2/2 (100%) | 6/9 (66.7%) | 0.217 |
|  | **REBOA (n=83)** | **RT (n=202)** |  |
| ***Brenner 2018*** |  |  |  |
| Post-occlusion SBP (mean/sd) mmHg | 89 (65) | 30 (51) | P<0.001 |
| Duration of aortic occlusion, min, median IQR | 31 (57) | 19 (21) | 0.002 |

## **Failure/success of REBOA technique**

Two studies reported the number of subjects in which the technique was successfully advanced.

**Table 8.** Number of success of REBOA technique.

| **Successful aortic occlusion** | **REBOA n (%)** |
| --- | --- |
| Brenner 2018 | 78/83 (94) |
| Dubose 2016 | 42/46 (91.3) |

# **Supplement D**. Internal validity

|  | Selection | | | | Comparability | Outcome | | |  |
| --- | --- | --- | --- | --- | --- | --- | --- | --- | --- |
| Cohort study | **Representativeness of the exposed cohort** | **Selection of the non exposed cohort** | **Ascertainment of exposure** | **Demonstration that outcome of interest was not present at start of study** | **Comparability of cohorts on the basis of the design or analysis** | **Assessment of outcome** | **Was follow-up long enough for outcomes to occur** | **Adequacy of follow up of cohorts** | **tot** |
| Abe 2016 | * | *  drawn from the same community as the exposed cohort | *  secure record | *  yes | *  study controls for important factor (propensity score matching) | *  record linkage: objective outcomes | §  Not reported | *  Subjects lost to follow up unlikely to introduce bias | **Good quality** |
| Aso 2017 | * | *  drawn from the same community as the exposed cohort | *  secure record | *  yes | *  study controls for important factor (propensity score matching) | *  record linkage: objective outcomes | *  Yes (28 days) | *  Subjects lost to follow up unlikely to introduce bias (30-40% for BMI) | **Good quality** |
| Brenner 2018 | *  somewhat representative of the average population in the community | *  drawn from the same community as the exposed cohort | *  secure record | *  yes | no control for confounding performed (similar baseline data) | *  record linkage: objective outcomes | §  Not specified  (longer than 24 hours) | §  Not reported | **Fair quality** |
| Dubose 2016 | * | *  drawn from the same community as the exposed cohort | *  secure record | *  yes | Unclear | *  record linkage: objective outcomes | *  Yes (24 h) | §  Not reported | **Good quality** |
| Garcia 2020 | * | drawn from the same community as the exposed cohort | *  secure record | *  yes | *  study controls for important factor (propensity score matching) | *  record linkage: objective outcomes | §  Not reported | §  Not reported | **Fair quality** |
| Inoue 2016 | * | *  drawn from the same community as the exposed cohort | *  secure record | *  yes | *  study controls for important factor (propensity score matching) | *  record linkage: objective outcomes | §  Not reported | §  Not reported | **Good quality** |
| Joseph 2015 | * | *  drawn from the same community as the exposed cohort | *  secure record | *  yes | *  study controls for important factor (propensity score matching) | *  record linkage: objective outcomes | *  Yes (24-hour  mortality, and mortality after 24 hours in both groups) | *  Missing treated as missing completely at random. | **Good quality** |
| Moore 2015 | * | *  drawn from the same community as the exposed cohort | *  secure record | *  yes | no control for confounding performed however authors declared no differences between groups at baseline | *  record linkage: objective outcomes | §  Not reported | §  Not reported | **Fair**  **quality** |
| Matsumara 2017 | * | *  drawn from the same community as the exposed cohort | *  secure record | *  yes | no control for confounding performed | *  record linkage: objective outcomes | Yes  (24 hours,30 days) | §  Not reported | **Fair quality** |
| Nori 2015 | * | *  drawn from the same community as the exposed cohort | *  secure record | *  yes | *  study controls for important factor (propensity score matching) | *  record linkage: objective outcomes | §  Not reported | *  excluded patients with missing survival  data | **Good quality** |
| Yamamoto 2019 | * | *  drawn from the same community as the exposed cohort | *  secure record | *  yes | *  study controls for important factor (propensity score matching) | *  record linkage: objective outcomes | Yes  (28 days, 90 days) | *  excluded patients with missing survival  data | **Good quality** |

§ Outcomes may have been influenced by time.

**Thresholds for converting the Newcastle-Ottawa scales to AHRQ standards (good, fair, and poor):**

Good quality: 3 or 4 stars in selection domain AND 1 or 2 stars in comparability domain AND 2 or 3 stars in outcome/exposure domain

Fair quality: 2 stars in selection domain AND 1 or 2 stars in comparability domain AND 2 or 3 stars in outcome/exposure domain

Poor quality: 0 or 1 star in selection domain OR 0 stars in comparability domain OR 0 or 1 stars in outcome/exposure domain

**Note**: A study can be awarded a maximum of one star for each numbered item within the Selection and Outcome categories. A maximum of two stars can be given for Comparability

# **Supplement E**. Summary of Findings Tables

| **REBOA compared to control (open surgery/no-REBOA) for hemorrage** | | | | | |
| --- | --- | --- | --- | --- | --- |
| **Outcomes** | **№ of participants  (studies) Follow up** | **Certainty of the evidence (GRADE)** | **Relative effect (95% CI)** | **Anticipated absolute effects** | |
|  |  |  |  | **Risk with control (open surgery/no-REBOA)** | **Risk difference with REBOA** |
| Overall Crude Mortality | 5819 (11 observational studies) | ⨁◯◯◯ VERY LOW ^a,b,c^ | **OR 0.68** (0.38 to 1.22) | 507 per 1.000 | **95 fewer per 1.000** (226 fewer to 49 more) |
| Overall Crude Mortality-subgroup analysis: REBOA vs open surgery | 1657 (5 observational studies) | ⨁◯◯◯ VERY LOW ^b^ | **OR 0.42** (0.32 to 0.54) | 852 per 1.000 | **144 fewer per 1.000** (204 fewer to 95 fewer) |
| Overall Crude Mortality-subgroup analysis: REBOA vs RT+REBOA | 106 (1 observational study) | ⨁◯◯◯ VERY LOW ^b,d,e^ | **OR 0.13** (0.04 to 0.47) | 900 per 1.000 | **361 fewer per 1.000** (635 fewer to 91 fewer) |
| Overall Crude Mortality-subgroup analysis: REBOA vs no-REBOA | 4056 (5 observational studies) | ⨁◯◯◯ VERY LOW ^b,f^ | **OR 1.68** (1.03 to 2.72) | 419 per 1.000 | **129 more per 1.000** (7 more to 243 more) |
| Overall Adjusted Mortality | 5819 (8 observational studies) | ⨁◯◯◯ VERY LOW ^a,b,c^ | **OR 0.87** (0.48 to 1.58) | 507 per 1.000 | **35 fewer per 1.000** (177 fewer to 112 more) |
| Overall Adjusted Mortality-subgroup analysis: REBOA vs open surgery | 1657 (5 observational studies) | ⨁⨁◯◯ LOW ^b^ | **OR 0.38** (0.20 to 0.74) | 852 per 1.000 | **166 fewer per 1.000** (317 fewer to 42 fewer) |
| Overall Adjusted Mortality-subgroup analysis: REBOA vs RT+REBOA | 106 (1 observational study) | - | not estimable | 900 per 1.000 | **900 fewer per 1.000** (900 fewer to 900 fewer) |
| Overall Adjusted Mortality-subgroup analysis: REBOA vs no-REBOA | 4056 (5 observational studies) | ⨁◯◯◯ VERY LOW ^a,b^ | **OR 1.40** (0.79 to 2.46) | 419 per 1.000 | **83 more per 1.000** (56 fewer to 221 more) |
| Overall adjusted mortality ED | 2573 (3 observational studies) | ⨁◯◯◯ VERY LOW ^a,b,c^ | **OR 0.80** (0.14 to 4.74) | 167 per 1.000 | **29 fewer per 1.000** (140 fewer to 320 more) |
| Overall adjusted mortality 24 h | 534 (2 observational studies) | ⨁◯◯◯ VERY LOW ^b,c,f^ | **OR 1.00** (0.10 to 9.48) | 259 per 1.000 | **0 fewer per 1.000** (225 fewer to 509 more) |
| Overall adjusted mortality 1 month | 654 (2 observational studies) | ⨁◯◯◯ VERY LOW ^b,c^ | **OR 0.77** (0.36 to 1.61) | 234 per 1.000 | **44 fewer per 1.000** (135 fewer to 96 more) |
| ***The risk in the intervention group** (and its 95% confidence interval) is based on the assumed risk in the comparison group and the **relative effect** of the intervention (and its 95% CI).   **CI:** Confidence interval; **OR:** Odds ratio | | | | | |
| **GRADE Working Group grades of evidence** **High certainty:** We are very confident that the true effect lies close to that of the estimate of the effect **Moderate certainty:** We are moderately confident in the effect estimate: The true effect is likely to be close to the estimate of the effect, but there is a possibility that it is substantially different **Low certainty:** Our confidence in the effect estimate is limited: The true effect may be substantially different from the estimate of the effect **Very low certainty:** We have very little confidence in the effect estimate: The true effect is likely to be substantially different from the estimate of effect | | | | | |

#### Explanations

a. I^2^>90%

b. variability of setting (no-prehospital)

c. Confidence intervals crossed the line of no difference with plausible effects in favor to the experimental group

d. risk of bias in outcome assessment and follow-up

e. number of events <200

f. I^2^>75%
